# Development of *Protium heptaphyllum* essential oil-loaded nanocapsules: experimental design and biological activity

**DOI:** 10.3762/bjnano.17.67

**Published:** 2026-07-28

**Authors:** Debora Freitas Silva, Kaidy Giselle Orellana, Allessya Lara Dantas Formiga, Jin Wang, Eloisa Helena de Aguiar Andrade, Aline Collares Valente Pinheiro, Maria Izabel de Jesus, Francisco Canindé Ferreira de Luna, Wallax Augusto Silva Ferreira, Edivaldo Herculano Correa de Oliveira, Francisco Humberto Xavier Junior, Emmanuel Abraham Ho, Marcele Fonseca Passos

**Affiliations:** 1 Institute of Biological Sciences, Federal University of Pará, PA, Brazil; 2 Technological Development Group on Biomaterials and Bioproducts of the Amazon, PA, Brazil; 3 Laboratory for Drug Delivery and Biomaterials, School of Pharmacy, University of Waterloo, ON, Canada; 4 Postgraduate Program in Bioactive Natural and Synthetic Products, Federal University of Paraíba, PB, Brazil; 5 Laboratory of Pharmaceutical Biotechnology, Department of Pharmaceutical Sciences, Center of Health Sciences, Federal University of Paraíba, PB, Brazil; 6 Adolpho Ducke Laboratory, Botany Coordination, Museu Paraense Emílio Goeldi, Belém, PA, Brazil; 7 Environmental Health Laboratory, Environment Section, Evandro Chagas Institute, PA, Brazil; 8 Laboratory of Cytogenomics and Environmental Mutagenesis, Environment Section, Evandro Chagas Institute, PA, Brazil

**Keywords:** biocompatibility, cytokine modulation, drug delivery systems, nanoprecipitation, wound healing

## Abstract

Poly(ε-caprolactone) nanocapsules containing breu branco (*Protium heptaphyllum*) essential oil were successfully prepared by nanoprecipitation and optimized using a Box–Behnken design. Spherical nanosystems were obtained, with a mean particle size of 172.10 ± 0.90 nm, a polydispersity index of 0.14 ± 0.04, a zeta potential of −25.49 ± 1.08 mV and an encapsulation efficiency above 99%, in addition to long-term colloidal stability. Cell viability assays in HaCaT cells showed high biocompatibility, with cell viability above 70% up to 6 mg/mL and only moderate toxicity at 12 mg/mL. In antimicrobial assays, the free oil had no inhibitory effect against *Staphylococcus aureus*, while the nanocapsules exhibited a minimum inhibitory concentration of 0.55 mg/mL, indicating enhanced antibacterial activity. Wound-healing assays further demonstrated improved keratinocyte migration compared to serum-free controls. Additionally, cytokine profiling by cytometric bead array indicated an anti-inflammatory profile, with strong suppression of IL-6 and selective increases in IL-2, IL-4, and TNF-α at higher doses (6 mg/mL) in nanocapsule-treated fibroblasts. Overall, these findings demonstrate that nanoencapsulation not only stabilizes breu branco essential oil but also enhances its antimicrobial, regenerative, and immunomodulatory effects, while fostering the sustainable valorization of Amazonian biodiversity.

## Introduction

The biodiversity of the Amazon basin provides a vast source of natural bioactive compounds that have attracted increasing interest for their therapeutic properties and applications in pharmacology, cosmetology, and biotechnology [1–2]. Studying these compounds supports the development of novel natural products and promotes the sustainable use of renewable biological resources with high therapeutic potential. Among these, essential oils are particularly important due to their complex chemical composition, rich in terpenoids and phenolic compounds known to exhibit antimicrobial, anti-inflammatory, and antioxidant activities [3–4]. These properties underscore the importance of Amazonian resources for both sustainable use and the development of innovative delivery strategies.

The essential oil of *Protium heptaphyllum* (*P. heptaphyllum*) resin, commonly known as breu branco, is a bioactive natural product of significant pharmacological interest. Phytochemical analyses show a high concentration of monoterpenes, particularly *p*-cymene [5–6], which contribute to its antimicrobial, anti-inflammatory, and wound-healing properties [7]. These biological activities make breu branco essential oil (EO) a promising candidate for therapeutic and cosmetic formulations. However, its practical use in industry is limited by physicochemical issues such as high volatility, poor solubility, and susceptibility to oxidative degradation [8]. Overcoming these limitations requires innovative stabilization and delivery methods that preserve bioactivity and allow for scalability.

In this context, nanotechnology provides a promising solution by enabling the encapsulation of bioactive compounds within polymeric systems. Nanoencapsulation of monoterpene-rich essential oils such as oregano, thyme, and tea tree have been widely studied, demonstrating improved physicochemical stability, enhanced antimicrobial activity, and controlled release profiles [2,9–10]. This approach protects the active constituents from degradation and enhances bioavailability [11–12].

Polymeric nanocapsules are especially advantageous, providing increased stability as well as ensuring controlled release and improved interactions with biological matrices [13–14]. They can be engineered using various techniques, such as emulsification–solvent evaporation, interfacial polymerization, solvent displacement, and microfluidics, each with specific advantages and limitations depending on the desired application [15–16]. Among these, nanoprecipitation (NP) is notable for producing uniform particles through a low-cost, reproducible process based on polymer nucleation in aqueous media [17–18]. Previous work, such as that by Vauthier et al. [15], successfully demonstrated oil-loaded nanocapsules using interfacial polymerization, supporting the importance of evaluating critical parameters like particle size, polydispersity index (PDI), and zeta potential.

Building on this evidence, the present study aimed to develop and characterize polymeric nanocapsules containing breu branco essential oil using the NP method. Despite the extensive literature on the nanoencapsulation of monoterpene-rich essential oils, studies specifically addressing *Protium heptaphyllum* remain scarce. In particular, the influence of its chemical profile, predominantly composed of α-pinene and *p*-cymene, on biological responses such as cytokine modulation and wound healing has not been systematically investigated. Previous studies have primarily focused on its chemical composition and biological activities in the free form, with limited exploration of its behavior when incorporated into nanostructured delivery systems.

Therefore, this study aims to develop and optimize polymeric nanocapsules containing *P. heptaphyllum* essential oil using a Box–Behnken experimental design, focusing on key physicochemical parameters such as particle size, PDI, and zeta potential. The optimized nanosystems were subsequently evaluated in terms of stability, encapsulation efficiency, cell viability, antimicrobial activity against *Staphylococcus aureus* (*S. aureus*), wound healing potential (scratch assay), and cytokine modulation. The results of this study contribute to a better understanding of the physicochemical and biological behavior of *P. heptaphyllum* essential oil when nanoencapsulated. Thus, this work provides a basis for future investigations involving various applications, while fostering the sustainable valorization of Amazonian biodiversity.

## Results and Discussion

### Chemical composition of *P. heptaphyllum* essential oil

The chromatographic analysis of *P. heptaphyllum* essential oil revealed the presence of 31 distinct compounds, with a predominance of monoterpenes, accounting for 92.1% of the total composition (Table 1). The principal constituent identified was *p*-cymene (56.7%), a monoterpene with broad-spectrum bioactivity, including antimicrobial, anti-inflammatory, antioxidant, vasorelaxant, antinociceptive, antidiabetic, antiviral, and mitochondrial-targeting effects [19–20]. Its high concentration suggests a central role in the biological activities attributed to the essential oil (EO). Studies have demonstrated that *p*-cymene modulates opioid pathways and calcium channels, mechanisms associated with inflammatory and neuropathic pain [21], and inhibits the growth of *Aspergillus flavus* at concentrations as low as 80 mg/L [22].

Other major monoterpenes included *p*-mentha-1(7),8-diene (9.54%), recognized for its insect repellent activity [23], and α-pinene (7.54%), widely known to exhibit anti-inflammatory, antioxidant, antimicrobial, antitumor, neuroprotective, and hepatoprotective effects through multiple molecular pathways [24–29].

**Table 1:** Chemical composition of *P. heptaphyllum* essential oil.

Components			

Monoterpenes	RI_Lit_^a^	RI_calc_^b^	%

α*-*thujene	923	928	0.67
α-pinene	932	935	7.54
camphene	947	948	1.02
sabinene	974	972	0.41
3-*p*-menthene	1014	982	6.44
menthomenthene	1014	1001	5.00
*p*-mentha-1(7),8-diene	1014	1006	9.54
α*-*terpinene	1016	1015	2.20
*p*-cymene	1023	1026	56.70
*p*-cymenene	1088	1089	0.08
γ-terpinene	1057	1055	1.86
terpinolene	1087	1083	0.52
*trans*-calamenene	1088	1088	0.80

Oxygenated monoterpenes			

terpinen-4-ol	1174	1179	1.55
*p*-cymen-8-ol	1175	1185	0.24
α-terpineol	1191	1193	0.25
camphor	1138	1145	0.11

Aromatic aldehydes			

cuminaldehyde	1238	1240	0.08
phellandral	1275	1276	0.05

Phenols			

thymol	1290	1297	0.05

Sesquiterpenes			

α-cubebene	1348	1344	0.17
α-copaene	1377	1372	0.38
(*E*)-caryophyllene	1419	1415	0.20
β-humulene	1442	1440	0.06
germacrene D	1485	1479	0.02
γ-cadinene	1513	1509	0.07
δ-cadinene	1523	1514	0.17

Oxygenated sesquiterpenes			

junenol		1618	0.08

^a^RI_calc_: Retention time index calculated; ^b^RI_lit_: Retention time index from NIST and [30].

Despite their lower abundance, the presence of sesquiterpene hydrocarbons, such as α-cubebene (0.17%), α-copaene (0.38%), (*E*)-caryophyllene (0.20%), and δ-cadinene (0.17%), are notable for their well-known anti-inflammatory and immunomodulatory activities, which may act synergistically with monoterpenes [31–32]. Oxygenated compounds, including terpenic alcohols, ketones, and aromatic aldehydes, were detected in low concentrations, but should not be disregarded, given their potential contribution to the oxidative stability and sensorial properties of the oil.

### Formulation optimization

Table 2 presents the Box–Behnken design matrix and the observed responses for *D*_h_, PDI, and zeta potential. These results reflect the combined effects of the independent variables, that is, polymer concentration (20–100 mg), EO content (50–150 mg), and surfactant concentration (20–60 mg) on the physicochemical properties of the nanocapsules.

**Table 2:** Relationship between independent and dependent variables. Values represent mean ± SD (*n* = 3). For more information, see Table 5 below.^a^

Experiment	PCL	EO	TWEEN 80	*D*_h_ (nm)	PDI	Zeta potential (mV)

2	1	−1	0	447.90 ± 6.40	0.05 ± 0.06	−30.94 ± 0.62
10	0	1	−1	298.30 ± 8.30	0.20 ± 0.03	−31.89 ± 0.72
11	0	−1	1	245.20 ± 4.50	0.12 ± 0.03	−26.47 ± 0.36
15	0	0	0	266.10 ± 0.70	0.11 ± 0.02	−24.99 ± 0.20
3	−1	1	0	220.60 ± 4.50	0.13 ± 0.03	−32.52 ± 0.76
12	0	1	1	358.00 ± 3.80	0.23 ± 0.01	−18.84 ± 0.47
5	−1	0	−1	239.20 ± 6.00	0.16 ± 0.06	−30.14 ± 0.18
9	0	−1	−1	289.00 ± 2.80	0.13 ± 0.01	−27.97 ± 1.05
14	0	0	0	264.90 ± 4.20	0.13 ± 0.03	−24.81 ± 1.31
13	0	0	0	260.90 ± 3.50	0.15 ± 0.04	−25.35 ± 1.68
4	1	1	0	473.10 ± 11.00	0.08 ± 0.10	−27.81 ± 0.82
7	−1	0	1	186.10 ± 3.00	0.09 ± 0.01	−27.97 ± 5.34
8	1	0	1	396.30 ± 10.90	0.14 ± 0.02	−29.64 ± 0.86
6	1	0	−1	305.80 ± 13.80	0.13 ± 0.04	−32.55 ± 1.86
1	−1	−1	0	172.10 ± 0.90	0.14 ± 0.04	−25.49 ± 1.08

^a^*D*_h_: hydrodynamic diameter; PDI: polydispersity index.

The hydrodynamic diameter *D*_h_ varied substantially across formulations, ranging from 172.10 ± 0.90 to 473.10 ± 11.00 nm, indicating a strong influence of formulation variables on particle size. The smallest particles were obtained in Experiment 1 (PCL −1; EO −1; Tween 80 0), whereas higher polymer levels were consistently associated with increased diameters. The quadratic model demonstrated satisfactory explanatory and predictive performance (*R*^2^ = 90.78%, adjusted *R*^2^ = 87.71%, predicted *R*^2^ = 82.44%) (Supporting Information File 1, Table S1). The lack-of-fit test was not significant (*p* = 0.492) (Supporting Information File 1, Table S2) with an adequate precision of 18.91. This indicates that the models adequately describe the experimental data. Furthermore, TEM analysis was performed to confirm the morphology and structural integrity of the nanocapsules prior to optimization and can be seen in Figure S3 (Supporting Information File 1). Assays revealed predominantly spherical morphology and smooth surfaces.

**Figure 1:**
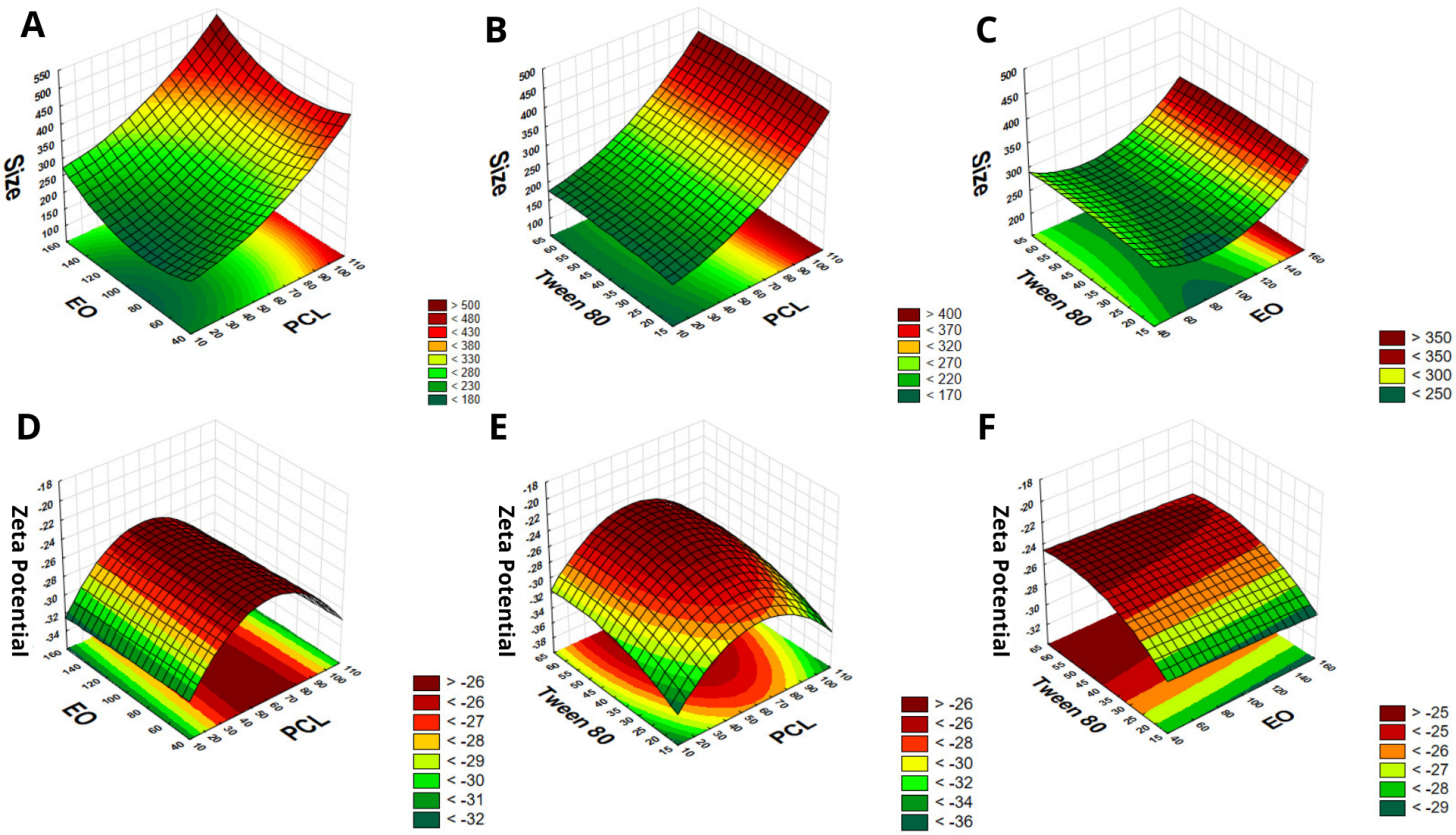
3D plots showing the relationship between independent variables on particle size (A–C) and zeta potential (D–F) of nanocapsules containing *P. heptaphyllum* essential oil. EO: concentration of *P. heptaphyllum* essential oil.

The results indicate that PCL concentration was the primary factor influencing particle size, exhibiting significant linear and quadratic effects (*p* < 0.05). Increasing polymer concentration may have elevated the viscosity of the organic phase, potentially reducing solvent diffusion during nanoprecipitation and delaying nucleation processes. Such diffusion-limited conditions are commonly associated with enhanced droplet coalescence prior to stabilization, which can contribute to larger particle formation [33–34]. The presence of a significant quadratic term suggests a non-linear dependence, indicating a possible balance between nucleation and growth at higher PCL levels. According to the Stokes–Einstein relationship, increased viscosity reduces the molecular diffusion coefficient, supporting this mechanistic explanation [35–36]. EO concentration also showed a significant effect on *D*_h_ (*p* < 0.001), including a relevant quadratic contribution (Table 3). This is because higher oil content may increase the internal core volume and modify interfacial characteristics, potentially favoring droplet growth and particle enlargement. Analysis of the response surface plots (Figure 1) further suggests that the combined increase in PCL and EO may reduce the efficiency of mass transport during nanoprecipitation, consistent with diffusion-controlled mechanisms described by the Fickian diffusion model [37].

**Table 3:** Regression coefficients and statistical significance of the fitted quadratic models (s: significant terms).

Term	*D*_h_ (nm)		PDI		Zeta potential (mV)	

	Coef.	*p*-Value	Coef.	*p*-Value	Coef.	*p*-Value

Constant	264	0^a^	0.13	0^a^	−25.05	0^a^
PCL	100.63	0^a^	−0.01	0.10	−0.60	0.16
EO	24.49	0^a^	0.02	0.01^a^	−0.02	0.95
Tween 80	6.67	0.29	−0.00	0.65	2.46	0^a^
PCL * PCL	24.33	0.013^a^	−0.03	0.02^a^	−3.96	0^a^
EO * EO	40.10	0^a^	0.00	0.75	−0.18	0.77
Tween 80 * Tween 80	−6.45	0.49	0.03	0.02^a^	−1.06	0.09^a^
PCL * EO	−5.83	0.51	0.00	0.47	2.54	0^a^
PCL * Tween 80	35.92	0^a^	0.01	0.17	0.18	0.76
EO * Tween 80	25.86	0^a^	0.00	0.50	2.88	0^a^

^a^*p* < 0.05.

**Figure 2:**
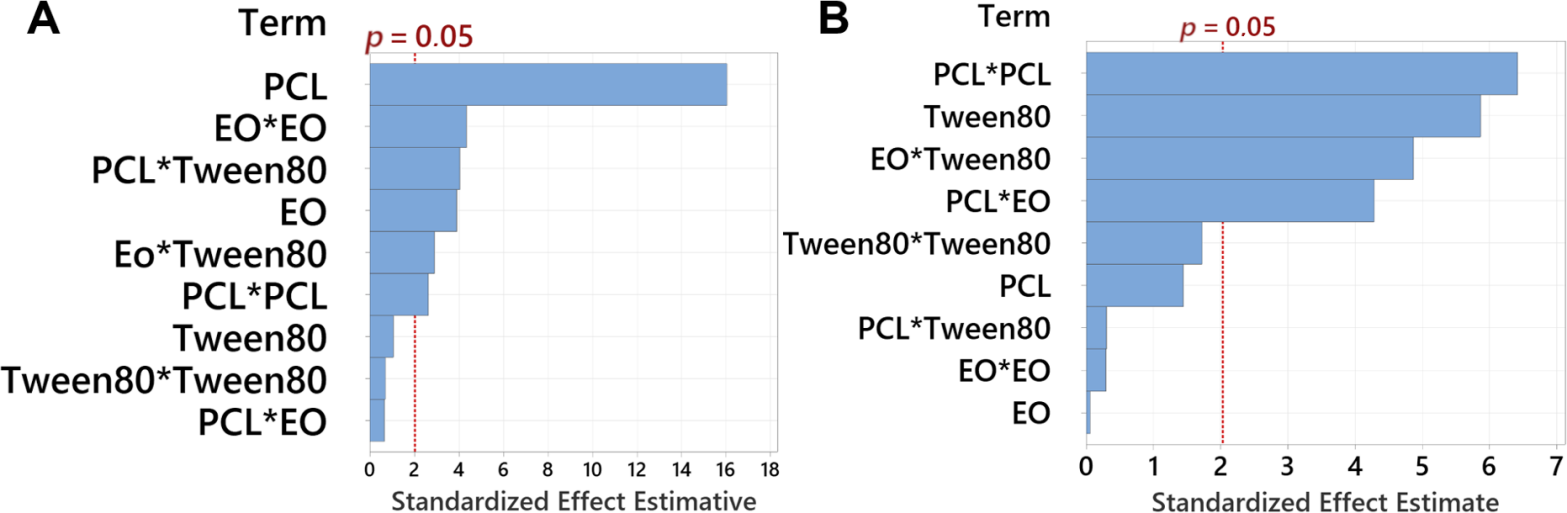
Pareto charts represent the influence of independent variables and their interactions on (A) the hydrodynamic diameter of the nanocapsules and (B) the zeta potential. Bars exceeding the dashed line indicate statistically significant effects (*p* < 0.05), according to ANOVA analysis.

Regarding the zeta potential, the model showed moderate explanatory capacity (*R*^2^ = 78.82%, adjusted *R*^2^ = 71.76%) and a predictive performance (predicted *R*^2^) of 58.32% (Supporting Information File 1, Table S1). Although lower than those for *D*_h_, these values are satisfactory for interfacial properties, which are inherently influenced by dynamic surface phenomena such as surfactant rearrangement and charge distribution at the nanoparticle interface [16]. The lack-of-fit test was not significant (*p* = 0.078), confirming adequate model specification (Supporting Information File 1, Table S2). Residual analysis did not reveal systematic deviations (Supporting Information File 1, Figure S2), supporting the statistical robustness of the model. Tween 80 was the most influential linear factor for the zeta potential (Figure 2) (*p* < 0.001), indicating its dominant role in modulating surface charge through steric and electrostatic stabilization mechanisms. Significant interaction terms (PCL * EO and EO * Tween 80) further suggest that surface organization depends on the balance between polymer matrix composition and surfactant coverage [38].

In contrast, the PDI model exhibited limited predictive capability (*R*^2^ = 47.91%, adjusted *R*^2^ = 30.54%, predicted *R*^2^ = 1.18%) (Supporting Information File 1, Table S1), suggesting that this response was not strongly influenced by the formulation variables within the studied range. This behavior is consistent with the nature of PDI, which is often affected by particle formation dynamics and measurement variability rather than solely by formulation composition. Nevertheless, the model remained statistically significant, while the lack-of-fit test was not significant (*p* = 0.139) (Supporting Information File 1, Table S2), indicating that it is not structurally invalid, but was not used for the optimization. Importantly, all PDI values remained below 0.3, indicating homogeneous systems and relatively narrow size distributions across all formulations. Therefore, PDI was considered as a qualitative indicator of dispersion homogeneity rather than a primary parameter for optimization. Under such low-variability conditions, small experimental fluctuations and instrumental sensitivity can disproportionately affect the coefficient of determination, mathematically reducing *R*^2^_pred_ despite adequate model structure. Residual diagnostics showed no systematic patterns (Supporting Information File 1, Figure S3), suggesting that deviations arise from experimental variability rather than model misspecification.

Overall, the combined statistical analyses confirm that particle size was the parameter that was most responsive to formulation changes and therefore represented the primary optimization criterion. Among all designs, Experiment 1 yielded the most favorable formulation, with *D*_h_ = 172.10 ± 0.90 nm, PDI = 0.14 ± 0.04, and zeta potential = −25.49 ± 1.08 mV (Table 2). This optimized nanocapsule, designated as NPNC, was selected for subsequent biological assays.

### Encapsulation efficiency

The calibration curve exhibited strong linearity (*R*^2^ = 0.995; *y* = 40.298*x* − 0.3835) within the evaluated concentration range. The encapsulation efficiency (EE%) of the NPNC nanocapsules reached 99.94 ± 0.1%, indicating minimal loss of the active compound during the preparation and purification process. The high encapsulation efficiency observed is consistent with the lipophilic nature of essential oil components (70% of lipophilic constituents), which exhibit strong affinity for the oily core of polymeric nanocapsules. Such compatibility enhances solubilization in the organic phase during nanoprecipitation, resulting in near-complete incorporation. Efficiencies exceeding 95% have been reported in polymeric systems containing lipophilic essential oils [39–40], reinforcing the stability and reproducibility of the process. However, the ultrafiltration-based method may overestimate encapsulation efficiency because free oil can be retained within the filtration system.

### Physical stability of nanocapsules

**Figure 3:**
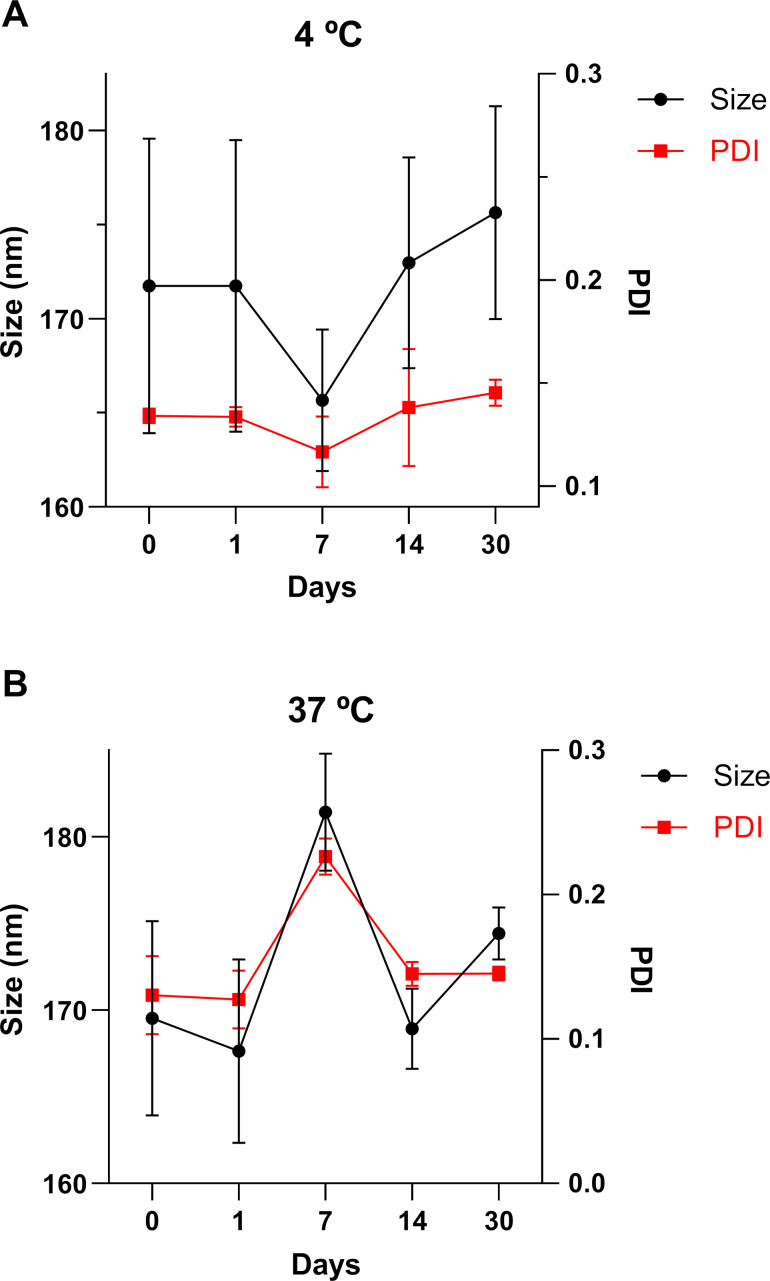
Physical stability of polymeric suspensions as a function of storage temperature over 30 days. (A) Hydrodynamic diameter (Size, nm) and polydispersity index (PDI) of nanoparticles stored at 4 °C. (B) Hydrodynamic diameter and PDI of nanoparticles stored at 37 °C. Measurements were performed at predetermined time points (0, 1, 7, 14, and 30 days). Data are presented as mean ± standard deviation (SD) (*n* = 3). Statistical analysis was performed using one-way ANOVA followed by Dunnett’s multiple comparisons test to compare each time point against the initial condition (day 0). Statistical significance is indicated as *p* < 0.05.

Figure 3 shows the physical stability of the NPNCs. The nanocapsules exhibited uniform particle size distributions, with PDI values consistently below 0.3, indicating high homogeneity and colloidal stability across the evaluated temperature conditions [41]. Maintaining stable particle size is critical, as variations in this parameter can influence surface area, alter diffusion, and affect release kinetics, ultimately compromising the pharmacokinetic performance of the formulation [42]. The sustained low PDI values throughout the entire period further confirm the absence of aggregation or coalescence, thereby preserving the structural integrity of the nanocapsules [43–44].

The observed stability may be attributed to the physicochemical characteristics of the nanocapsules, including their colloidal organization and the properties of the polymeric shell. Additionally, the unchanged particle size over time, even under thermal stress, indicate that no phase separation, leakage of the encapsulated compound, or interfacial degradation occurred during storage [45].

### Cell viability

**Figure 4:**
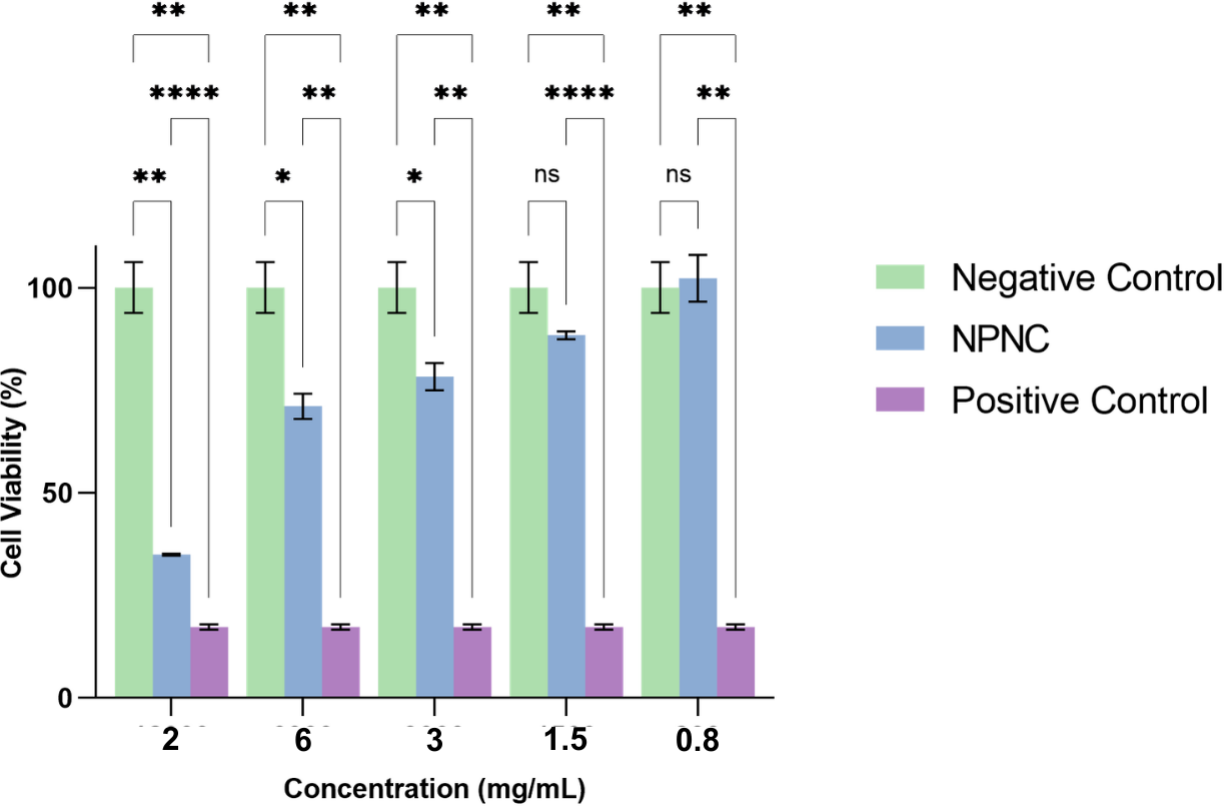
Cell viability (%) of HaCaT cells after exposure to nanocapsules containing *P. heptaphyllum* essential oil (NPNC) at different concentrations (mg/mL). Cells were treated for the indicated concentrations, and viability was assessed relative to untreated controls. Data are presented as mean ± standard deviation (SD) (*n* = 3). Negative control corresponds to cells cultured in DMEM, while positive control represents cells treated with 10% DMSO in DMEM. Statistical analysis was performed using one-way ANOVA followed by Dunnett’s multiple comparisons test. Statistical significance is indicated as *p < 0.05, **p < 0.01, ***p < 0.001, and ****p < 0.0001; ns, not significant.

The cell viability following treatment with NPNC (Figure 4) showed a clear dose-dependent effect, with higher concentrations of encapsulated oil reducing viability in immortalized human keratinocyte epithelial cells (HaCat). At 12 mg/mL, a marked decrease was observed, consistent with previous reports demonstrating the toxicity of essential oils at elevated levels.

This effect may be associated with the presence of lipophilic monoterpenes, including *p*-cymene and α-pinene, which have been reported to interact with phospholipid bilayers, potentially altering membrane organization and permeability [46–47]. Future studies must clarify the underlying mechanisms by using specific assays to evaluate membrane integrity, assess oxidative stress, or determine apoptosis.

According to ISO 10993-5 guidelines, a viability reduction greater than 30% compared to controls indicates cytotoxicity. In HaCaT keratinocytes, *P. heptaphyllum* essential oil-loaded nanocapsules maintained viability above 70% at all tested concentrations up to 6 mg/mL, supporting their biocompatibility within this range.

### Antimicrobial activity

**Figure 5:**
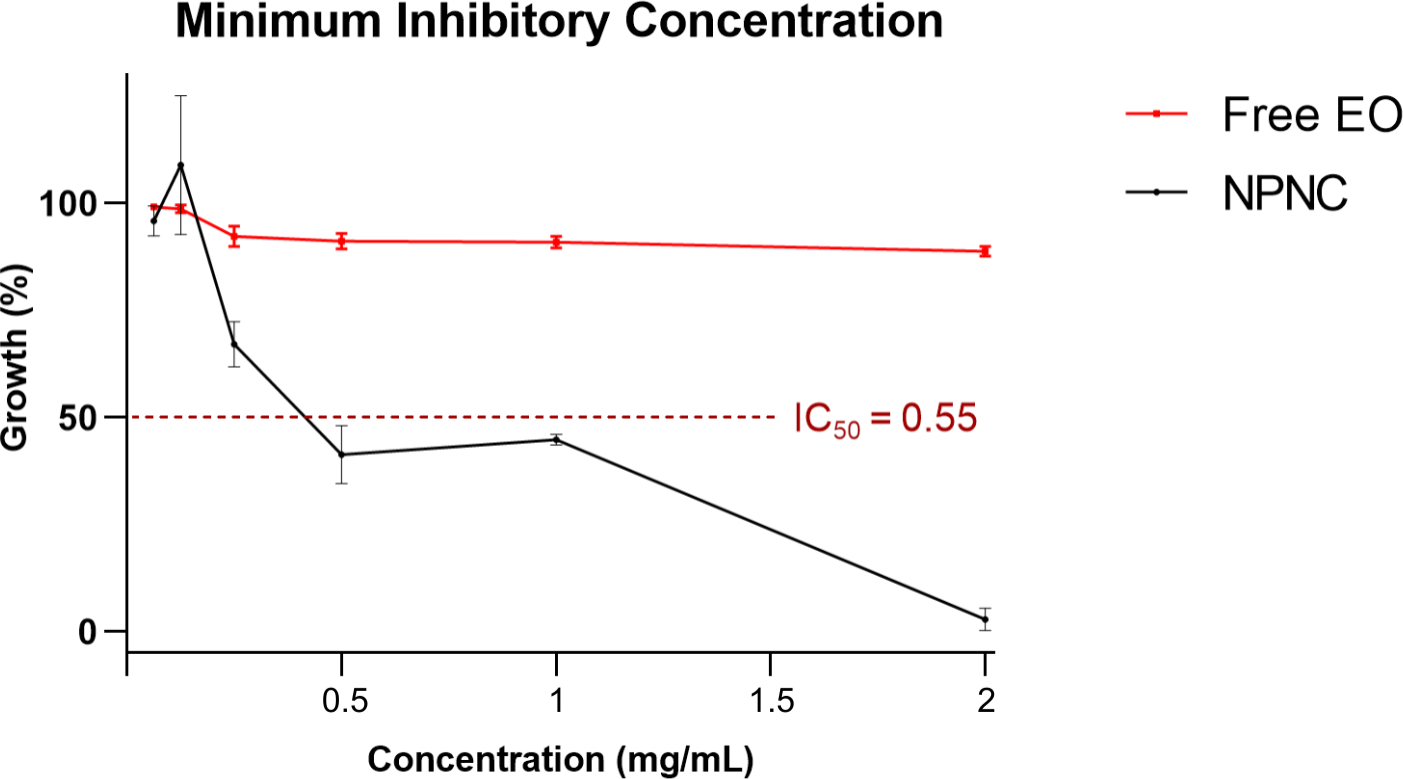
Antimicrobial activity against *Staphylococcus aureus* (ATCC 25923) following exposure to free *P. heptaphyllum* essential oil (Free EO) and nanocapsules containing the oil (NPNC) at increasing concentrations (mg/mL). Bacterial growth (%) was determined relative to untreated controls. Data are presented as mean ± standard deviation (SD) (*n* = 3). The dashed red line indicates the 50% inhibitory concentration (IC_50_), estimated at 0.55 mg/mL. Statistical analysis was performed using two-way ANOVA followed by Sidak’s multiple comparisons test to evaluate differences between formulations (Free EO vs NPNC) across concentrations. Statistical significance is indicated as *p* < 0.05.

Antimicrobial activity was evaluated exclusively against *S. aureus*, a Gram-positive bacterium widely associated with skin and wound infections. Nanocapsules containing *P. heptaphyllum* essential oil (NPNC) exhibited significant antimicrobial activity against *S. aureus* (ATCC 25923), with a minimum inhibitory concentration (MIC) of 0.55 mg/mL (IC 95%: 0.55–56 mg/mL) (Figure 5). In contrast, the free EO exhibited no significant antibacterial activity against the same strain, even at higher concentrations. This result is consistent with findings from de Lima et al. [48], who reported low activity of non-encapsulated *P. heptaphyllum* oil against *S. aureus*.

The increased activity observed after nanoencapsulation supports the fact that nanostructuring improves the physicochemical performance of the EO. Specifically, encapsulation reduces volatility, enhances aqueous dispersion, and promotes stronger interaction with bacterial surfaces. Similar results were reported by Granata et al. [49], who observed a decrease in the MIC of oregano oil when formulated in nanocapsules, highlighting the contribution of nanotechnology in improving the antimicrobial potential of natural compounds. This effect is mechanistically linked to the increased surface-to-volume ratio of nanoparticles, which favors direct bacterial contact and may contribute to sustained release of active constituents [50]. The release profile of such systems is influenced by factors such as polymer composition, shell thickness, and interactions between the encapsulated compound and the carrier matrix.

Additionally, the effective concentration remains within a biologically safe range, as demonstrated in HaCaT cells, ensuring antimicrobial activity without compromising cell viability. Taken together, these findings provide evidence that the nanocapsule system enhances the antibacterial efficiency of *P. heptaphyllum* oil while maintaining cell viability, supporting its potential as a candidate for biomedical and pharmaceutical applications.

### Migration

**Figure 6:**
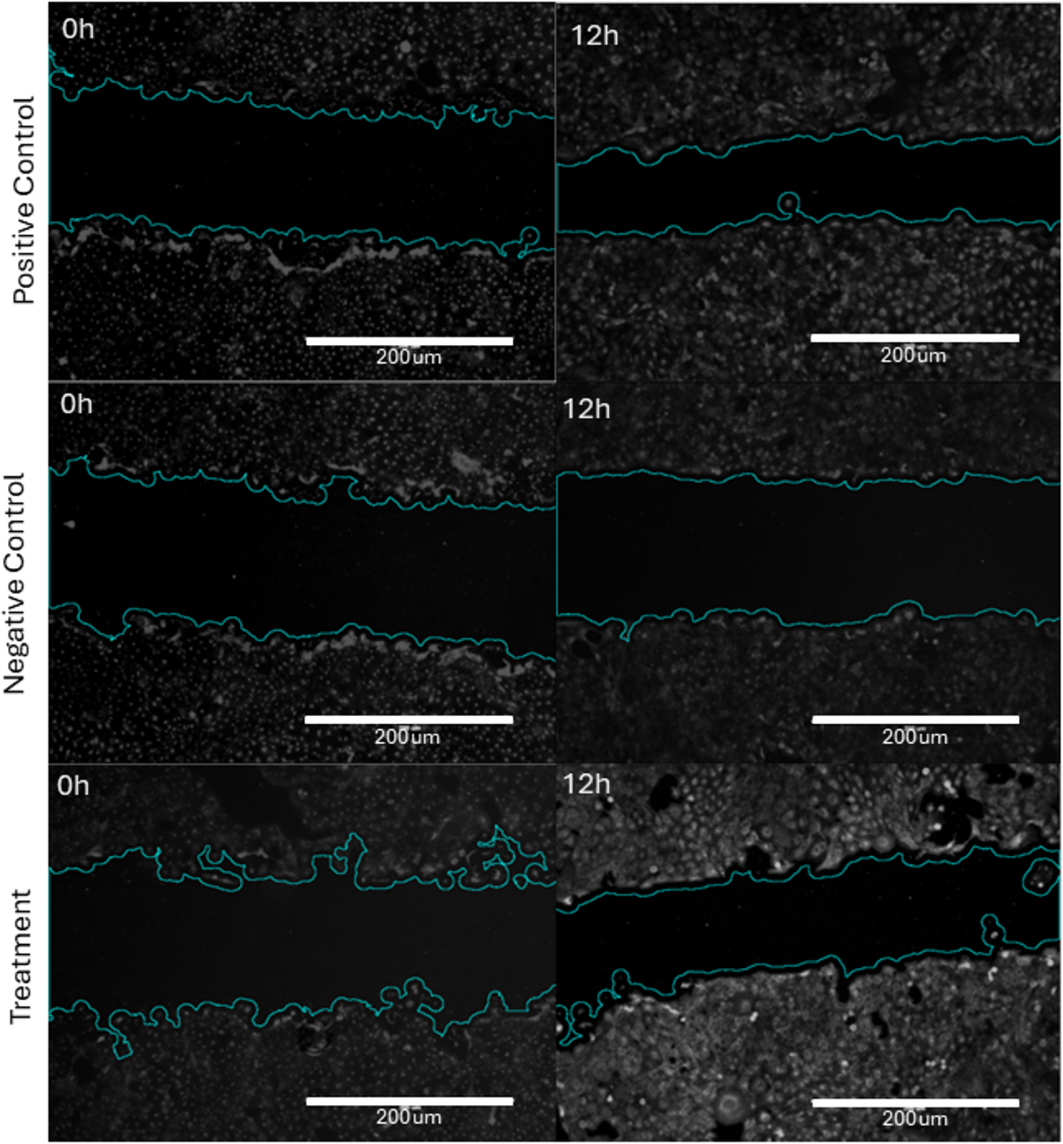
(A) Scratch-wound assay performed on HaCaT monolayers cultured under three conditions: medium control (DMEM + 10% FBS), negative control (DMEM without FBS), and NPNC treatment (1:8 dilution). Images were captured at 5× magnification using an EVOS FL Auto Imaging System microscope (Thermo Fisher Scientific, Kitchener, Canada) at 0 h and 12 h after incubation. Green lines indicate the wound edges at each time point. Scale bar: 200 μm. (B) Quantification of wound closure percentage at 12 h.

The in vitro wound healing potential of NPNC, using a scratch wound model, is illustrated in Figure 6. The results showed that exposure of HaCaT cells to NPNC significantly accelerated the scratch gap closure compared to controls. After 12 h, the FBS-supplemented positive control reached 31.8 ± 5.4% closure, while the serum-free negative control showed only 7.1 ± 4.2% closure. The NPNC group exhibited intermediate closure (16.8 ± 8.8%) (Table 4), indicating enhanced migratory activity compared to serum-deprived conditions. This effect likely results from intrinsic nanocarrier features, including greater dispersion and colloidal stability of encapsulated molecules, which together enhance local bioavailability. Notably, *P. heptaphyllum* essential oil is rich in *p*-cymene, a terpene primarily known for its antimicrobial and anti-inflammatory actions rather than direct wound repair. However, nanoencapsulation may enhance its regenerative effects by reducing microbial interference [51], modulating inflammatory responses [48], and improving membrane delivery through increased solubility and lipophilicity [52]. These combined effects provide a mechanistic rationale for the moderate but significant improvement in keratinocyte migration observed with the nanocapsule treatment.

**Table 4:** Effect of nanocapsules containing *P. heptaphyllum* essential oil (NPNC) on wound closure in HaCaT cells. Wound closure (%) was evaluated after 12 h for positive control (medium supplemented with FBS), negative control (medium without FBS), and cells treated with NPNC (1:8 dilution). Data are presented as mean ± standard deviation (SD) (*n* = 3). Statistical analysis was performed using one-way ANOVA followed by Dunnett’s multiple comparisons test. Statistical significance is indicated as *p* < 0.05.

Wound closure (%)			

Time (h)	Medium with FBS	Medium without FBS	NPNC

0	0	0	0
12	31.8 ± 5.4	7.1 ± 4.2	16.8 ± 8.8

### Cytokine dosage by cytometric bead array

**Figure 7:**
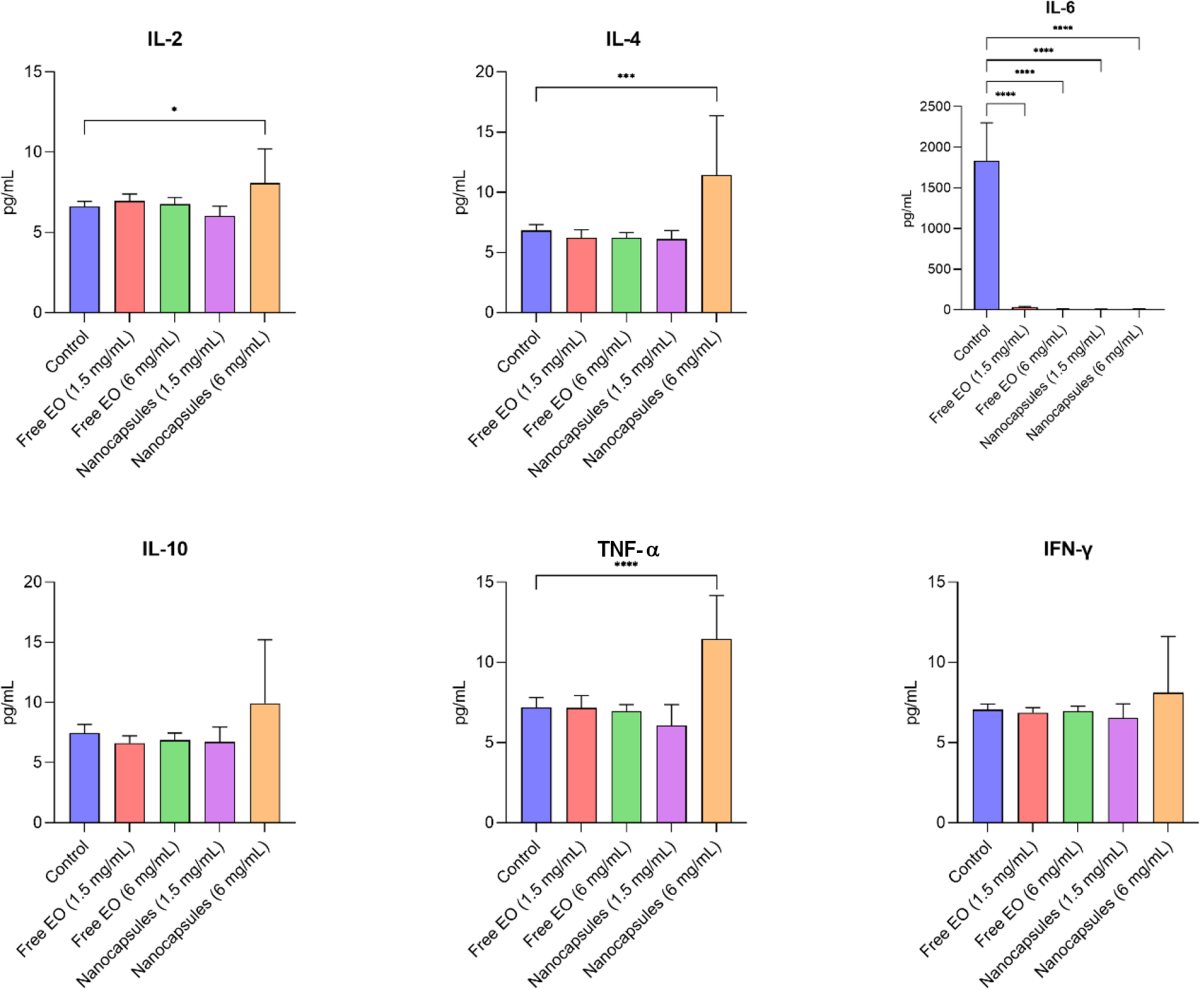
Effect of *P. heptaphyllum* essential oil (EO) and EO-loaded polymeric nanocapsules (NPNC) on cytokine secretion by MRC-5 fibroblasts. Cells were treated with free EO (1.5 or 6 mg/mL) or NPNC (1.5 or 6 mg/mL) for 24 h, and culture supernatants were analyzed for IL-2, IL-4, IL-6, IL-10, TNF-α, and IFN-γ concentrations (pg/mL) using the BD™ CBA Human Th1/Th2/Th17 Cytokine Kit. Bars represent mean ± SD (*n* = 8 independent replicates). Statistical comparisons between treated group and the untreated control were performed by one-way ANOVA followed by Dunnett's post hoc test. *p < 0.05, ***p < 0.001, ****p < 0.0001.

The cytometric bead array (CBA) results showed that *P. heptaphyllum* EO, especially in its nanoencapsulated form, selectively modulated the cytokine profile in MRC-5 fibroblasts in a concentration-dependent manner (Figure 7). The most consistent result was a marked reduction in IL-6 levels in all treated groups compared to the control.

The IL-6 suppression was observed at both free and nanoencapsulated concentrations, indicating potential anti-inflammatory effect, which may reflect the activity of monoterpenes present in the EO, particularly *p*-cymene and α-pinene. However, the precise intracellular mechanisms require further investigation through pathway-specific assays, such as NF-κB activation and gene expression profiling. At the highest NPNC concentration (6 mg/mL), TNF-α levels increased significantly, while lower concentrations had no effect. TNF-α has well-documented dual roles: Moderate concentrations promote tissue repair by stimulating fibroblast proliferation and extracellular matrix remodeling, whereas excessive or sustained production leads to chronic inflammation, cytotoxicity, and impaired wound healing [53–54]. The observed TNF-α increase may indicate either early reparative activation or dose-dependent cellular stress from increased bioactive compound delivery via nanocapsules. Future dose-optimization studies should determine the threshold at which TNF-α shifts from reparative to detrimental. Simultaneously, IL-4 increased only at the highest NPNC concentration (6 mg/mL). IL-4 is a known promoter of M2 macrophage polarization and tissue repair processes [55–56]. The simultaneous increase of TNF-α and IL-4 at the highest dose suggests complex, dose-dependent immunomodulation rather than unidirectional effects, warranting mechanistic investigation to determine whether these changes support tissue repair or inflammatory activation. IL-2 secretion increased significantly only at 6 mg/mL NPNC. While IL-2 primarily drives T cell proliferation in adaptive immunity [57–58], its role in fibroblasts is poorly characterized. This dose-dependent increase may reflect immunostimulation facilitated by enhanced intracellular delivery through polymeric nanocapsules. Future studies should determine whether IL-2 upregulation leads to functional outcomes such as increased proliferation or matrix production. IL-10 and IFN-γ levels remained unchanged under all conditions. The stability of IL-10 suggests that neither EO nor NPNC suppresses the endogenous anti-inflammatory capacity of fibroblasts. Similarly, the absence of IFN-γ changes suggest no excessive type-1 immune activation under the tested conditions.

Nanoencapsulation may enhance the bioavailability and potential sustained release of EO compounds, which in turn could lead to higher concentrations in target tissues and prolonged exposure, potentially explaining the increased cytokine responses observed at higher doses in our study [59–61]. Some studies report that nanoencapsulated EOs reduce pro-inflammatory cytokines (IL-6 and TNF-α) in wound healing and arthritis models while improving therapeutic outcomes [59–60]. The consistent suppression of IL-6 at all concentrations observed in our study supports anti-inflammatory effects, while dose-dependent increases in TNF-α, IL-4, and IL-2 at the highest concentration suggest a complex immunomodulatory profile that requires careful interpretation. The biological significance of these changes cannot be determined from cytokine quantification alone. Further functional and mechanistic studies are necessary to fully characterize the immunological implications of these formulations.

## Conclusion

Nanocapsules of *P. heptaphyllum* EO were successfully produced by nanoprecipitation, achieving high encapsulation efficiency (>99%), structural stability, and uniform spherical morphology. These formulations maintained cell viability in keratinocytes (>70%), with reduced viability only at excessively high concentrations. Nanoencapsulation improved antibacterial activity and reduced *S. aureus* inhibition concentration, compared to the free oil, and promoted in vitro wound closure, highlighting its potential for tissue repair. CBA showed selective immunomodulatory effects, with strong suppression of IL-6 and selective increases in IL-2, IL-4, and TNF-α at higher doses (6 mg/mL) in nanocapsule-treated fibroblasts, suggesting an anti-inflammatory profile that supports tissue regeneration while minimizing nonspecific inflammation. Together, these findings establish nanoprecipitation as an effective and scalable strategy to engineer polymeric nanosystems that stabilize Amazonian oils while enhancing their therapeutic and immunomodulatory properties.

### Limitations and future perspectives

The chemical composition of EOs can vary significantly depending on factors such as geographic origin, harvest season, and environmental conditions, which may directly influence their biological properties. Variations in the relative abundance of key monoterpenes in branco breu EO, such as α-pinene and *p*-cymene, may affect biological responses, including antimicrobial activity and cytokine modulation. Therefore, the reproducibility and generalizability of the observed effects often depend on the specific chemical profile of the raw material used. Future investigations should incorporate multiple batches and establish correlations between chemical composition and biological activity to strengthen the translational relevance of the research.

In vitro release kinetics studies are highly relevant because, without them, a direct correlation between physicochemical properties and biological responses cannot be determined. Specifically, correlating release behavior with both antimicrobial and cytokine responses is crucial to fully elucidating the mechanism of action of nanocapsules. Future studies should directly address this correlation gap to advance the field, using dialysis membrane methods under physiological conditions to evaluate the release kinetics.

Further investigations should examine the antimicrobial activity of nanocapsules against a wider range of microorganisms, particularly Gram-negative strains such as *Escherichia coli* and *Pseudomonas aeruginosa* due to their outer membranes that have reduced susceptibility to hydrophobic compounds like EOs. This approach would enable a more comprehensive evaluation of the antimicrobial potential of the nanocapsules.

## Experimental

### Materials and methods

*P. heptaphyllum* EO was purchased from a commercial supplier (Citroleo^®^, Brazil) and accompanied by a certificate of analysis. All experiments were performed using a single batch, ensuring internal consistency across the experimental analyses. The research was registered in the National System for the Management of Genetic Heritage and Associated Traditional Knowledge (SisGen) under registration number A4D47E3.

Polycaprolactone (PCL) (*M*_w_ ≈ 14,000, CAS 24980-41-4), Span^®^ 80, Amicon^®^ Ultra-15 centrifugal filters (100 kDa cutoff), tryptic soy broth (dehydrated culture medium), PKH26 kit, acetonitrile (≥99.9%, CAS 75-05-8), DMSO, and acetone (≥99.9%, CAS 67-64-1) were obtained from Millipore Sigma (Kitchener, Canada). Tween^®^ 80 was purchased from Medisca (Kitchener, Canada), and 0.22 μm polyethersulfone filters were obtained from Montreal Biotech (Kitchener, Canada). Dulbecco’s Modified Eagle Medium (DMEM, 320-030-CL) was supplied by Wisent (QC, CA), and fetal bovine serum (FBS) was purchased from Gibco (New York, USA). The MTS (3-(4,5-dimethylthiazol-2-yl)-5-(3-carboxymethoxyphenyl)-2-(4-sulfophenyl)-2*H*-tetrazolium) cell proliferation assay (CellTiter 96^®^ Aqueous One Solution) was acquired from Promega (Kitchener, Canada). The HaCaT cell line was obtained from Cytion (Eppelheim, Germany), the human normal fetal lung fibroblasts (MRC-5) were acquired from the Rio de Janeiro Cell Bank (BCRJ), and the *S. aureus* (ATCC 25923) strain was supplied by Cedarlane Labs (Kitchener, Canada).

### Composition of *P. heptaphyllum* essential oil

The chemical composition of the volatile compounds in *P. heptaphyllum* EO was determined by gas chromatography coupled with mass spectrometry (GC/MS) using a Shimadzu QP Plus-2010 system (Belém, Brazil). The equipment was fitted with a DB-5MS capillary silica column (30 m length, 0.25 mm and 0.25 µm film thickness) (Agilent Technologies, Inc.).

Helium was used as carrier gas at a constant linear velocity of 36.5 cm/s. Samples were injected in splitless mode (split ratio 0:1), with an injection volume of 2 µL of EO diluted in hexane (2 µL in 1 mL). The injector temperature was set at 250 °C. The oven temperature was programmed from 60 to 250 °C at a rate of 3 °C/min.

The ion source and other components were maintained at 220 °C, and the quadrupole scanned from 39 to 500 Da at a rate of one scan per second, using electron impact ionization at 70 eV. Volatile constituents were identified based on the calculation of the linear retention index (RI), obtained from the retention times of a homologous series of n-alkanes injected under the same conditions, along with analysis of the fragmentation patterns in the mass spectra, which were compared with reference data from system libraries and literature reports.

### Nanocapsule synthesis and experimental design

Nanocapsules were prepared using nanoprecipitation techniques. The organic phase consisted of *P. heptaphyllum* EO (50–150 mg), polycaprolactone (20–100 mg), and Span^®^ 80 (20 mg), solubilized in 6 mL of acetone. Simultaneously, the aqueous phase was prepared with 12 mL of a Tween^®^ 80 solution (20–60 mg) in Milli-Q^®^ water. Nanoparticles were formed by adding the organic phase to the aqueous phase using a syringe with an 11.2 mm diameter, under magnetic stirring at 400 rpm and room temperature. The dispersion was then concentrated in a rotary evaporator at 40 °C, with constant rotation at 100 rpm for 45 min, at 40 mbar. The resulting nanocapsules were washed with Milli-Q^®^ water and centrifuged in centrifugal filters at 3000*g* for 10 min at 4 °C to remove residual solvents. This step was repeated three times to ensure complete removal of residues, after which the nanoparticles were filtered using 0.22 μm polyethersulfone filters.

For nanomaterial optimization, a Box–Behnken design with three factors and three levels was used (Table 5), as described in [62]. The independent variables evaluated were PCL concentration (20–100 mg), EO concentration (50–150 mg), and Tween^®^ 80 surfactant concentration (20–60 mg), coded into 15 experiments with central point replicates (Table 1). The dependent variables analyzed were mean *D*_h_, PDI, and zeta potential.

**Table 5:** Variables and their levels for the Box–Behnken design.

Independent variable	LEVEL		

	−1	0	1

PCL (mg)	20	60	100
EO (mg)	50	100	150
Tween^®^ 80 (mg)	20	40	60

Optimal parameters were defined primarily based on minimizing the *D*_h_, which was considered the main optimization criterion. PDI and zeta potential were evaluated as secondary indicators of colloidal homogeneity and physicochemical stability, ensuring that the optimized formulation maintained low size dispersion and adequate surface charge for suspension stability.

Data processing and optimization of experimental conditions were carried out by constructing three-dimensional response surface plots and contour plots using Minitab^®^ 21.4 statistical software (State College, USA). The adequacy and predictive performance of the Box–Behnken models were assessed using determination coefficients (*R*^2^, adjusted *R*^2^, and predicted *R*^2^), analysis of variance (ANOVA), and residual diagnostics. Additionally, the experimental design was executed in random order, as described by Esmaeili and Gholami [63].

### Characterization of mean hydrodynamic size, polydispersity index, and zeta potential

The mean values of *D*_h_, PDI, and zeta potential of the nanoparticles were determined by dynamic light scattering (DLS) using a Zetasizer Nano-ZS^®^ (Malvern Instruments Ltd, Canada). All samples were previously diluted in Milli-Q^®^ ultrapure water at a ratio of 1:100 prior to analysis. For zeta potential measurements, a 0.05 mol/L KCl solution was used. Measurements were performed at 25 °C in triplicate.

### Determination of encapsulation efficiency (%)

Encapsulation efficiency (EE) was determined according to the methodology of Valencia et al. [40], using the ultrafiltration–centrifugation technique with adaptations. Since this method may be affected by adsorption or retention phenomena within the filtration membrane, Microcon^®^ devices with Ultracel^®^ regenerated cellulose membranes (low-binding, hydrophilic) were utilized to minimize nonspecific interactions with lipophilic compounds. Recovery tests were performed using free EO subject to the same filtration process.

The filtrate obtained after ultrafiltration (Amicon Ultra-0.5, Millipore^®^) of the nanocapsules was collected to quantify the non-encapsulated oil. The filtrate was diluted in acetonitrile (1:10 v/v), and its concentration was measured by UV–vis spectrophotometry (Cary 5000, Agilent, Waterloo, Canada). A calibration curve of the EO was constructed using serial dilutions prepared in acetonitrile within the analytical concentration range. Absorbance values were measured at 259 nm, previously determined as the wavelength of maximum absorption (λ_max_) following a full UV–vis spectral scan of the EO. The limit of detection (LOD ≈ 0.0016 mg/mL) and the limit of quantification (LOQ ≈ 0.005 mg/mL) were calculated according to ICH guidelines based on the standard deviation of the regression intercept and the slope of the calibration curve. Blank nanocapsules (polymer and surfactant without the EO and free EO) were subject to identical ultrafiltration and spectrophotometric analysis to exclude matrix interference. All analyses were carried out in triplicate. Encapsulation efficiency was calculated according to Equation 1:


[1] $$\text{EE}\left(\%\right)=\left(\frac{T-F}{T}\right)\times 100,$$

where EE is the encapsulation efficiency, *T* is the total amount of oil in the nanocapsule suspension, and *F* is the amount of free oil present in the filtrate after ultrafiltration.

### Study of the physical stability of optimized nanocapsules

Optimized nanocapsules were stored in airtight vials at 4 °C and 37 °C, protected from light, and analyzed at predetermined time points (0, 1, 7, 14, and 30 days). The mean particle size and PDI were monitored throughout the storage period [64]. Statistical analysis of physical stability data was performed using GraphPad Prism 9.0 (San Diego, USA). Data normality was assessed using the Shapiro–Wilk test prior to analysis. One-way ANOVA followed by Dunnett’s post hoc test was applied. Differences were considered statistically significant when *p* < 0.05.

### Cell viability assay

HaCaT cells were cultured in complete DMEM supplemented with 10% FBS and 1% penicillin–streptomycin and seeded in 96-well plates at a density of 5 × 10^4^ cells/well. Cells were incubated at 37 °C in a humidified incubator with 5% CO_2_ (air jacketed CO_2_ incubator basic, VWR). After 24 h, oil-loaded nanocapsules (NPNC) were added at concentrations ranging from 0.8 to 12 mg/mL and incubated for an additional 24 h. Subsequently, the treatment medium was removed, and 20 μL of MTS solution (Promega, Madison, WI, USA) was added to 180 μL of phenol red-free DMEM. Negative control cells were maintained in DMEM alone, while the positive control consisted of 10% (v/v) DMSO. Plates were incubated for 3 h (37 °C, 5% CO_2_), and absorbance was measured at 490 nm using a microplate reader (Varioskan LUX, Thermo Fisher Scientific, Kitchener, Canada), following the manufacturer’s instructions (Promega). All experiments were conducted in triplicate. Cell viability percentage was calculated according to Equation 2:


[2] $$\begin{array}{l} \text{relative cell viability}\left(\%\right)=\\ \frac{\text{absorbance of treatment}-\text{absorbance of blank}}{\text{absorbance of negative control}}\times 100 \end{array}$$

### Minimum inhibitory concentration determination

The MIC of *S. aureus* (ATCC 25923) was determined using the broth microdilution method in 96-well plates. Bacterial suspensions were prepared in tryptic soy broth and adjusted to a turbidity corresponding to the 0.5 McFarland standard (≈1.5 × 10^8^ CFU/mL). Serial dilutions were performed to test concentrations ranging from 0.062 to 2 mg/mL. Penicillin–streptomycin (20 µg/mL) was used as a positive control. The experiment was performed in triplicate. After incubation, the MIC was defined as the lowest concentration of the antimicrobial agent that visibly inhibited bacterial growth, as assessed by optical density (OD) at 600 nm using a microplate reader. Concentration–response data were log_10_-transformed and fitted using a four-parameter logistic nonlinear regression model in GraphPad Prism 9.0 (San Diego, USA) to determine the IC_50_. The IC_50_ value provides a quantitative measure of antimicrobial potency, complementing the MIC determination.

### Scratch wound assay

HaCaT epithelial cells were cultured in 24-well plates containing complete DMEM at an initial density of 5 × 10^4^ cells per well. Cells were incubated at 37 °C and 5% CO_2_ until they reached approximately 90% confluence. Prior to treatment, cells were maintained in serum-free medium for 12 h to induce nutrient deprivation. For the cell migration assay, an artificial gap (scratch) was created in the cell monolayer using a sterile 10 μL pipette tip. Cellular debris was removed by washing twice with PBS, and the culture medium was replaced with 1000 μL of the respective experimental, positive, or negative group solutions. Cells maintained in serum-free medium served as the negative control, while cells cultured in serum-supplemented medium (10% FBS) served as the positive control. After 12 h of treatment, cells were stained with the fluorescent dye PKH26 (16 μM) and incubated for 45 min. Excess dye was removed by washing with PBS, and adherent cells were retained for analysis. Cell migration was assessed by fluorescent imaging at 0 h and 12 h using an EVOS FL Auto Imaging System (Thermo Fisher Scientific, Kitchener, Canada) with a 5× objective. The scratch closure rate was analyzed by measuring the initial and final wound area using ImageJ software. Scratch closure percentage was calculated according to Equation 3:


[3] $$\text{scratch closure}\left(\%\right)=\frac{\text{initial area}-\text{final area}}{\text{initial area}}\times 100$$

### Cytokine quantification by cytometric bead array (CBA)

Human fibroblast MRC-5 cells were cultured in sterile flasks containing DMEM supplemented with 10% FBS, 100 U/mL penicillin, and 100 µg/mL streptomycin. Cultures were maintained at 37 °C in a humidified 5% CO_2_ atmosphere until suitable conditions were reached for the assay. For testing, cells were seeded in 96-well plates at a density of 1 × 10^4^ cells/well and incubated for 24 h to allow for adhesion. Cells were then treated with free *P. heptaphyllum* EO at concentrations of 1.5 and 6 mg/mL, as well as with polymeric nanocapsule-encapsulated oil at concentrations of 1.5 and 6 mg/mL. After 24 h of exposure under the same incubation conditions, culture supernatants were collected, thawed, and centrifuged at 300*g* for 5 min to remove cellular debris. Cytokine quantification (IL-2, IL-4, IL-6, IL-10, TNF-α, and IFN-γ) was performed using the BD Cytometric Bead Array (CBA) Human Th1/Th2 Cytokine Kit (BD Biosciences, San Jose, CA, USA), following the manufacturer’s instructions. Briefly, 50 µL of each sample supernatant was incubated with bead populations coated with cytokine-specific capture antibodies and PE-labeled detection reagent. Data acquisition was performed on a BD FACSCanto II flow cytometer (BD Biosciences), configured according to the manufacturer’s recommended parameters, acquiring at least 2100 events per bead region for each of the eight replicates per experimental group. Data analysis was conducted using FCAP Array v3.0 software (BD Biosciences), and cytokine concentrations were expressed in picograms per milliliter. The theoretical limits of detection were 3.7, 3.8, 4.5, 2.4, 4.9, and 2.6 pg/mL for IFN-γ, TNF-α, IL-10, IL-6, IL-4, and IL-2, respectively.

### Statistical analysis

Data obtained from in vitro assays were statistically analyzed using one-way or two-way analysis of variance (ANOVA), as appropriate, followed by suitable post hoc tests according to the experimental design and comparisons of interest. All analyses were performed using GraphPad Prism 9.0 (San Diego, USA). Data normality was assessed using the Shapiro–Wilk test. Results are presented as mean ± standard deviation (SD). Statistical significance was defined as follows: *p < 0.05, **p < 0.01, ***p < 0.001, and ****p < 0.0001; ns, not significant.

## Supporting Information

File 1 Additional experimental data.

## Data Availability

Data generated and analyzed during this study is available from the corresponding author upon reasonable request.
