# Supplementary material for: Development of Protium heptaphyllum essential oil-loaded nanocapsules: experimental design and biological activity
Source: Beilstein J Nanotechnol. 2026 Jul 28;17:974–90. doi: 10.3762/bjnano.17.67 (PMC13430529; doi:10.3762/bjnano.17.67)
Supplement: File 1 — Additional experimental data. [file Beilstein_J_Nanotechnol-17-974-s001.pdf]

BEILSTEIN JOURNAL OF NANOTECHNOLOGY

Supporting Information

for

Development of *Protium heptaphyllum* essential oil-loaded nanocapsules: experimental design and biological activity

Debora Freitas Silva, Kaidy Giselle Orellana, Allessya Lara Dantas Formiga, Jin Wang, Eloisa Helena de Aguiar Andrade, Aline Collares Valente Pinheiro, Maria Izabel de Jesus, Francisco Canindé Ferreira de Luna, Wallax Augusto Silva Ferreira, Edivaldo Herculano Correa de Oliveira, Francisco Humberto Xavier Junior, Emmanuel Abraham Ho and Marcele Fonseca Passos

Beilstein J. Nanotechnol. **2026,** *17,* 974–990. doi:10.3762/bjnano.17.67

Additional experimental data

License and Terms: This is a supporting information file under the terms of the Creative Commons Attribution License (https://creativecommons.org/licenses/by/4.0). Please note that the reuse, redistribution and reproduction in particular requires that the author(s) and source are credited and that individual graphics may be subject to special legal provisions.
The license is subject to the *Beilstein Journal of Nanotechnology* terms and conditions: (https://www.beilstein-journals.org/bjnano/terms)

Table S1: Model summary statistics for the fitted response surface model evaluating particle size. S: standard deviation of the regression (residual standard error); R^2: coefficient of determination; R^2 (adj): adjusted coefficient of determination; R^2 (pred): predicted coefficient of determination and adequate precision.

Model Summary					
	**S**	**R^2**	**R^2 (adj)**	**R^2 (pred)**	**Adeq. Precision**
**Size**	30.7290	90.78%	87.71%	82.44%	18.91
**PDI**	0.0445652	47.91%	30.54%	1.18%	7.46
**Zeta**	2.05155	78.82%	71.76%	58.32%	12.39

Table S2: Analysis of variance (ANOVA) for the fitted response surface model evaluating the effects of PCL, essential oil, and polysorbate concentration on the size. DF: degrees of freedom; Adj. SS: adjusted sum of squares; Adj. MS: adjusted mean square. Significant effects were determined at $p < 0.05$. The model was statistically significant, with no significant lack of fit.

Analysis of Variance (ANOVA) for Size					
**Source**	**DF**	**Adj. SS**	**Adj. MS**	**F-Value**	**P-Value**
Model	11	306780	27889	29.54	0.000
Blocks	2	147	74	0.08	0.925
Linear	3	258509	86170	91.26	0.000
PCL	1	243050	243050	257.39	0.000
EO	1	14391	14391	15.24	0.000
Tween 80	1	1068	1068	1.13	0.295
Quadratic	3	24212	8071	8.55	0.000
PCL * PCL	1	6557	6557	6.94	0.013
EO * EO	1	17816	17816	18.87	0.000
Tween 80 * Tween 80	1	460	460	0.49	0.490
Two-Factor Interaction	3	23912	7971	8.44	0.000
PCL * EO	1	408	408	0.43	0.515
PCL * Tween 80	1	15480	15480	16.39	0.000
EO * Tween 80	1	8024	8024	8.50	0.006
Error	33	31161	944		
Lack of Fit	27	25958	961	1.11	0.492

Pure Error	6	5203	867	*	*
Total	44	337941	–	–	–

Table S3: Analysis of variance (ANOVA) for the fitted response surface model evaluating the effects of PCL, essential oil, and polysorbate concentration on the PDI. DF: degrees of freedom; Adj. SS: adjusted sum of squares; Adj. MS: adjusted mean square. Significant effects were determined at $p < 0.05$. The model was statistically significant, with no significant lack of fit.

**Analysis of Variance (ANOVA) for PDI**					
**Source**	**DF**	**Adj. SS**	**Adj. MS**	**F-Value**	**P-Value**
Model	11	0.060272	0.005479	2.76	0.012
Blocks	2	0.006010	0.003005	1.51	0.235
Linear	3	0.020818	0.006939	3.49	0.026
PCL	1	0.005441	0.005441	2.74	0.107
EO	1	0.014955	0.014955	7.53	0.010
Tween 80	1	0.000422	0.000422	0.21	0.648
Quadratic	3	0.027610	0.009203	4.63	0.008
PCL * PCL	1	0.012843	0.012843	6.47	0.016
EO * EO	1	0.000200	0.000200	0.10	0.753
Tween 80 * Tween 80	1	0.012454	0.012454	6.27	0.017
Two-Factor Interaction	3	0.005834	0.001945	0.98	0.414
PCL * EO	1	0.001076	0.001076	0.54	0.467
PCL * Tween 80	1	0.003827	0.003827	1.93	0.174
EO * Tween 80	1	0.000931	0.000931	0.47	0.498
Error	33	0.065540	0.001986		
Lack of Fit	27	0.059975	0.002221	2.40	0.139
Pure Error	6	0.005564	0.000927	*	*
Total	44	0.125812	–	–	–

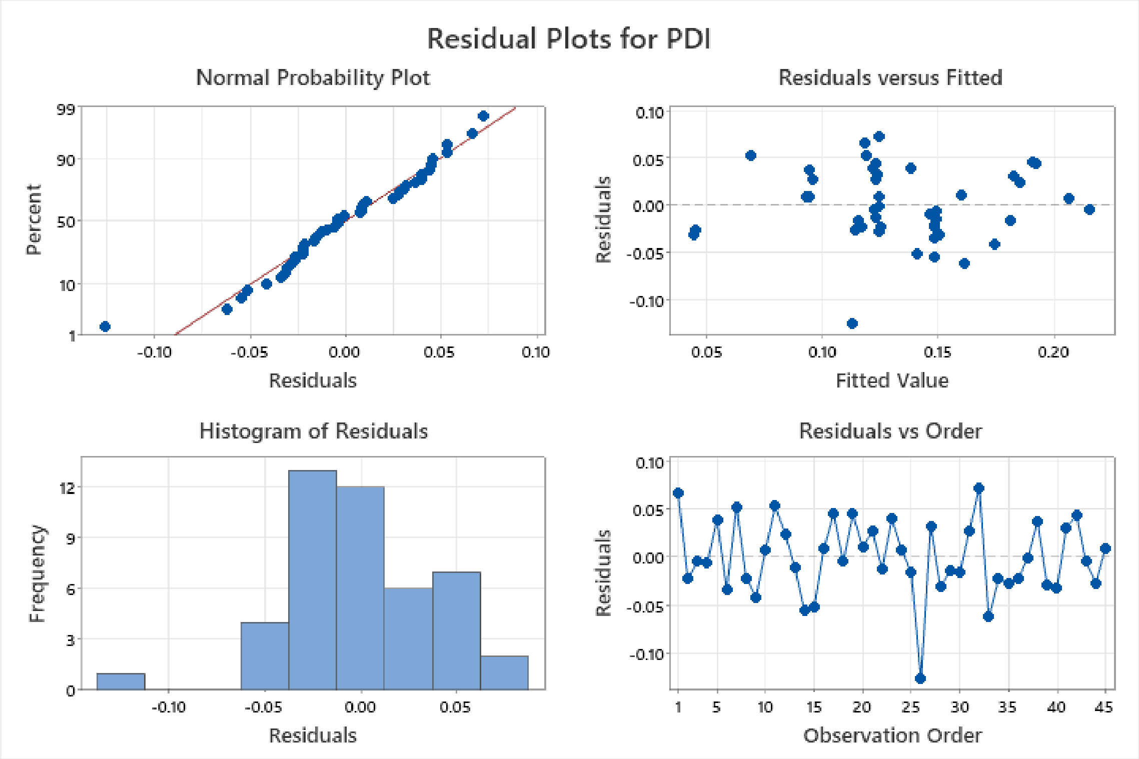

Figure S1: Residual PDI diagnostic plots for the fitted response surface models.

Table S4: Analysis of variance (ANOVA) for the fitted response surface model evaluating the effects of PCL, essential oil, and polysorbate concentration on the zeta potential. DF: degrees of freedom; Adj. SS: adjusted sum of squares; Adj. MS: adjusted mean square. Significant effects were determined at $p < 0.05$. The model was statistically significant, with no significant lack of fit.

Analysis of Variance (ANOVA) for Zeta Potential					
**Source**	**DF**	**Adj. SS**	**Adj. MS**	**F-Value**	**P-Value**
Model	11	516.804	46.982	11.16	0.000
Blocks	2	5.140	2.570	0.61	0.549
Linear	3	153.717	51.239	12.17	0.000
PCL	1	8.809	8.809	2.09	0.157
EO	1	0.014	0.014	0.00	0.955
Tween 80	1	144.894	144.894	34.43	0.000
Quadratic	3	180.315	60.105	14.28	0.000
PCL * PCL	1	173.533	173.533	41.23	0.000
EO * EO	1	0.361	0.361	0.09	0.771
Tween 80 * Tween 80	1	12.538	12.538	2.98	0.094
Two - Factor Interaction	3	177.632	59.211	14.07	0.000

PCL * EO	1	77.368	77.368	18.38	0.000
PCL * Tween 80	1	0.385	0.385	0.09	0.764
EO * Tween 80	1	99.879	99.879	23.73	0.000
Error	33	138.893	4.209		
Lack of Fit	27	129.730	4.805	3.15	0.078
Pure Error	6	9.162	1.527	*	*
Total	44	655.696	–	–	–

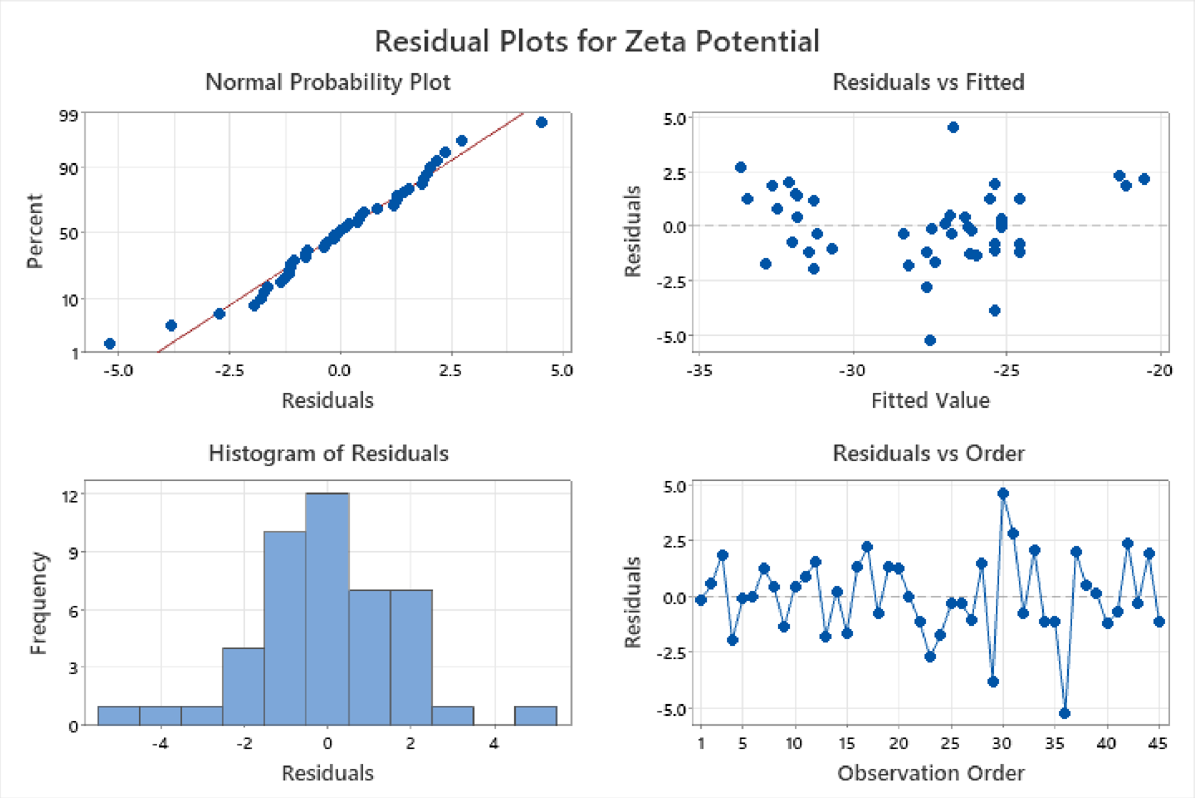

Figure S2: Residual zeta potential diagnostic plots for the fitted response surface models.

Morphology of nanocapsules

Experimental

Transmission electron microscopy (TEM) was performed to evaluate morphology and structural integrity of the nanocapsules prior to optimized formulation. Analyses were carried out using a ZEISS EM900 transmission electron microscope (Belém, PA, Brazil), equipped with a high-resolution MegaView 3G digital camera, operated at an acceleration voltage of 80 kV. Samples, both in their concentrated form and diluted at a 1:100 ratio in Milli-Q® water, were deposited onto carbon-coated copper grids and allowed to stand for 30 s. Non-adhered nanocapsules were removed using filter paper, and a drop of 2% phosphotungstic acid (pH 7.4) was applied as a negative stain for 30 s. Excess stain was removed with filter paper, and the grids were air-dried at room temperature before analysis, as described by Hedayati et al. [1].

This procedure was conducted in collaboration with the Evandro Chagas Institute (Belém, PA, Brazil).

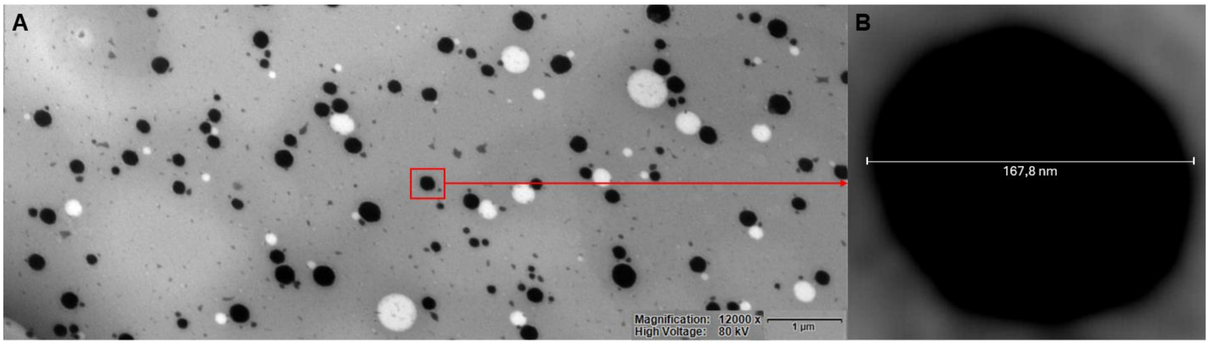

Figure S3: TEM images of NPNC obtained by nanoprecipitation. The image shows the overall particle distribution (magnification 12000× and 80 kV) (A), with a higher magnification highlighting a single nanocapsule (B).

References

1. Hedayati, S.; Niakousari, M.; Mohsenpour, Z. *Int. J. Biol. Macromol.* **2020,** *143,* 136–142. doi:10.1016/j.ijbiomac.2019.12.003
